# Genome sequence of white sturgeon herpesvirus 1 isolated from farmed white sturgeon (*Acipenser transmontanus*)

**DOI:** 10.1128/MRA.00571-23

**Published:** 2023-11-02

**Authors:** Thomas B. Waltzek, Kuttichantran Subramaniam, Andor Doszpoly, Joseph Hughes, Matej Vučak, Andrew J. Davison

**Affiliations:** 1 Department of Infectious Diseases and Immunology, College of Veterinary Medicine, University of Florida, Gainesville, Florida, USA; 2 HUN-REN Veterinary Medical Research Institute, Budapest, Hungary; 3 MRC-University of Glasgow Centre for Virus Research, Glasgow, United Kingdom; Katholieke Universiteit Leuven, Leuven, Belgium

**Keywords:** *Alloherpesviridae*, *Acipenser transmontanus*, sturgeon, genome

## Abstract

The genome sequence of white sturgeon herpesvirus 1, which was isolated from farmed white sturgeon (*Acipenser transmontanus*), was determined. Comparative analyses suggest the classification of this virus as a new species in a new genus in the family *Alloherpesviridae*.

## ANNOUNCEMENT

White sturgeon herpesvirus 1 (WSHV1) was isolated on the white sturgeon skin (WSSK-1) cell line from a juvenile white sturgeon experiencing elevated mortality on a commercial farm in California, USA, in 1991 (1). Additional herpesviruses were isolated subsequently from farmed white sturgeon experiencing elevated morbidity and mortality (2). Based on the differences from WSHV1 in serological properties and growth characteristics in cell culture, these isolates were designated white sturgeon herpesvirus 2 (WSHV2). Phylogenetic analyses based on 12 core genes present in all members of the family *Alloherpesviridae* revealed that WSHV1 is distantly related to WSHV2 (3) and that its closest relative is lake sturgeon herpesvirus (LSHV) (4).

The WSHV1 isolate (strain UC Davis) was passaged six times in WSSK-1 cells (1), and DNA was purified from the culture medium using a Blood & Cell Culture DNA mini kit (Qiagen). A sequencing library prepared using a Nextera XT kit (Illumina) was sequenced on a MiSeq instrument (Illumina) using a v3 600-cycle reagent kit, yielding 11,301,304 300 nt paired-end reads. The data were analyzed using default options, except where stated otherwise. The reads were trimmed using Trim Galore v.0.6.6 (https://www.bioinformatics.babraham.ac.uk/projects/trim_galore/) with the -q 25, --length 50, and --paired options. The trimmed reads were deduplicated using FastUniq v.1.1 (5) and subsampled using Seqtk v.1.3 (https://github.com/lh3/seqtk) with the sample, -s34, and 0.03 (to discard 97% of reads) options. The subsampled reads were assembled into contigs using SPAdes v3.14.1 (6) with the --careful, --cov-cutoff auto, and -k 33,77,127 options. Contigs related to the genomes of members of the family *Alloherpesviridae* were identified by using BLASTX (7) against the NCBI non-redundant protein database. These contigs were joined to others by extracting reads that extended their ends until overlaps were found. The genome termini were assigned as outlined below. The sizes of seven tandem reiterations were not resolved. The integrity of the sequence was verified by aligning the trimmed reads using Bowtie 2 v.2.3.1 (8) and Samtools v.1.3 (9) and inspecting the alignment using Tablet v.1.21.02.08 (10). The alignment incorporated 98% of reads at an average coverage depth of 14,704 reads/nt.

**Fig 1:**
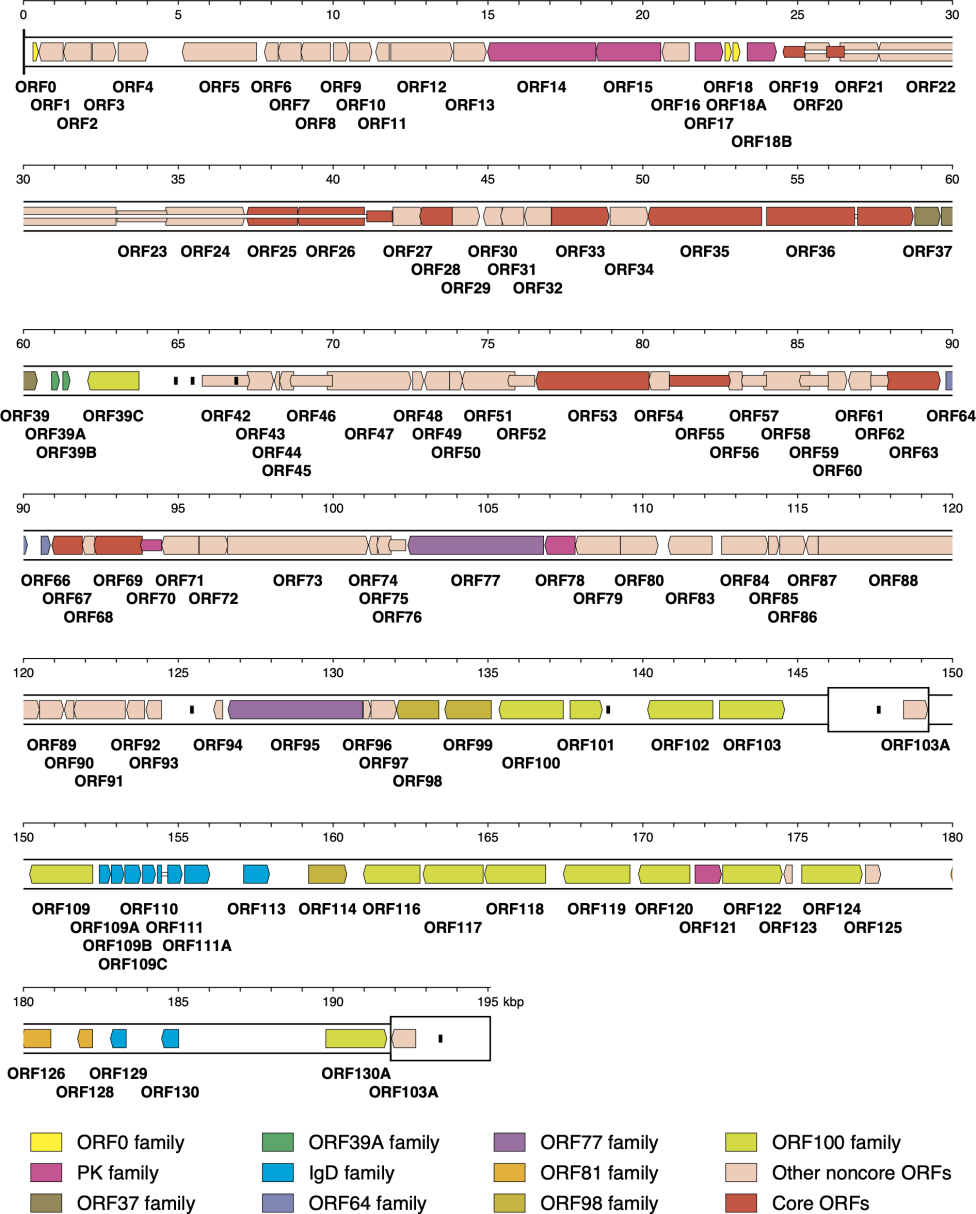
WSHV1 genome map. Unique regions (U_L_ and U_S_) are shown in a thinner format than inverted repeats (TR_L_/IR_L_ and IR_S_/TR_S_). Predicted functional ORFs are named to correspond with orthologs in the LSHV genome and are indicated by arrows colored according to the key as belonging to gene families (sets of paralogous genes), core ORFs (conserved among alloherpesviruses), and other noncore ORFs. Some ORFs are shown by narrow arrows to make their locations clearer. Introns connecting ORFs are shown as narrow white bars. Small, black rectangles indicate tandem repeats of undetermined size.

The WSHV1 genome (195,072 bp; 40% G + C) consists of a long unique region (U_L_; 145,962 bp) flanked by an inverted repeat (TR_L_/IR_L_; 26 bp), linked to a short unique region (U_S_; 42,630 bp) also flanked by an inverted repeat (TR_S_/IR_S_; 3,214 bp), yielding the overall structure TR_L_−U_L_−IR_L_−IR_S_−U_S_−TR_S_ (Fig. 1). The left terminus was assigned from a large set of reads sharing an end, and the right terminus from the adjacent sequence at the IR_L_-IR_S_ junction. A total of 129 open reading frames (ORFs) encoding functional proteins were predicted using approaches described previously (4) (Fig. 1).

Although WSHV1 and LSHV (4) are each other’s closest relatives, the core genes are only 76.8% identical. This suggests the creation of a new genus in the family *Alloherpesviridae* incorporating two new species. A second partially sequenced lake sturgeon herpesvirus (11) is more closely related to LSHV than WSHV1; the core genes are 84.3% identical.

## Data Availability

The WSHV1 genome sequence is available in GenBank under accession number OR001786. The sequence reads are available under BioProject accession number PRJNA1004073.
